# The Reaction to Diagnosis Questionnaire—Sibling Version: A Preliminary Study on the Psychometric Properties

**DOI:** 10.3390/ejihpe15080147

**Published:** 2025-07-29

**Authors:** Chiara Martis, Annalisa Levante, Flavia Lecciso

**Affiliations:** 1 Department of Human and Social Sciences, University of Salento, Via di Valesio, 73100 Lecce, Italy; annalisa.levante@unisalento.it (A.L.); flavia.lecciso@unisalento.it (F.L.); 2 Lab of Applied Psychology, Department of Human and Social Sciences, University of Salento, Via di Valesio, 73100 Lecce, Italy

**Keywords:** reaction to the diagnosis, RDQ-S, typically developing sibling, disability, measure, exploratory factor analysis, internal validity

## Abstract

Background: The diagnosis of a disability in a child may significantly impact the entire family system. While existing literature has primarily focused on parental reaction to the diagnosis, little is known about how typically developing siblings process this event. Methods: This exploratory study presented the preliminary psychometric properties of the Reaction to Diagnosis Questionnaire—Sibling Version, adapted from an instrument originally designed to assess parental reaction to the child’s diagnosis. Following a back-translation and adaptation process, a cross-sectional study was conducted on 623 typically developing siblings (*M* = 24.6 years, *SD* = 5.76) of individuals with neurodevelopmental disorders or physical disabilities. Results: Exploratory factor analyses supported a refined 32-item questionnaire with two factors—lack of resolution versus resolution, both showing excellent internal reliability. No significant differences were found based on typically developing sibling sex, age, or type of disability of the brother/sister, while correlational analyses indicated that greater disability severity was associated with lower resolution and higher lack of resolution. Conclusions: Results suggested that the Reaction to Diagnosis Questionnaire—Sibling Version is a promising tool for assessing the reaction to the brother/sister diagnosis on behalf of the typically developing siblings. Further research is needed to confirm these results and inform intervention programs promoting well-being and adaptive family functioning.

## 1. Introduction

Receiving a diagnosis of disability within a family, particularly when it concerns a child, can represent a stressful event that demands substantial emotional and psychological adjustment. For many families, it involves a reorganisation of their daily routines and roles, which can influence the quality of family relationships and the psychological well-being of each member (Sher-Censor & Shahar-Lahav, 2022).

Based on Bowlby’s Attachment Theory (Bowlby, 1982) and the Theory of Resolution of Loss and Trauma developed by Main and Hesse (1990), Marvin and Pianta (1996) proposed a theoretical framework in which the reaction to the diagnosis process of a child’s disability or chronic illness plays a central role for parents, as it is crucial to understanding the impact of the diagnosis on their psychological experience, on the management of the child with disability, and on the quality of the parent–child relationship. According to these authors (Marvin & Pianta, 1996), how parents respond to the child’s diagnosis is profoundly shaped by their internal working models, that is, the mental representations constructed over time regarding themselves, their child, and the parent–child relationship (Bowlby, 1982).

Existing literature suggested that the process of reacting to a diagnosis may unfold through a complex and dynamic sequence of emotional responses, potentially including shock, denial, intense emotional responses (e.g., anger, guilt, shame), and in more adaptive cases, a gradual progression toward resolution (Di Cagno et al., 1992; Kübler-Ross, 1970). Resolution entails a redefinition of parental expectations and a reorganisation of the parental identity and family life around the new reality, acknowledging both the child’s vulnerabilities and strengths. However, not all parents reach this final stage; parents who experience a lack of resolution remain stuck in the earlier phases, such as persistent denial or chronic anger, which may negatively affect the psychological well-being of the parent and the broader family system.

From this perspective, resolution is not merely a rational acknowledgement of the diagnosis but rather an emotional integration of the traumatic event into one’s narrative, allowing for a parent–child relationship that is attuned, responsive, and not distorted by unresolved emotions (Marvin & Pianta, 1996). Conversely, a lack of resolution reflects an internal narrative still dominated by confusion, pain, or rigid psychological defences, which impairs the parent’s ability to respond sensitively and coherently to the child’s needs (Marvin & Pianta, 1996).

While the process of parental reaction to the diagnosis has been extensively explored (Lecciso et al., 2013b; Marvin & Pianta, 1996; Milshtein et al., 2010; Sher-Censor & Shahar-Lahav, 2022), little is known about the typically developing (TD) siblings’ reaction to their brother/sister’s disability. Recently, however, scholars have increasingly turned their attention to this population, identifying it as potentially vulnerable but also rich in adaptive resources (e.g., Ashori, 2025; Kim et al., 2025; Lecciso et al., 2025b; Tomeny et al., 2017).

TD siblings often play a crucial role in both present and future lives of individuals with disability and face a unique adaptation process, marked by a complex interplay of challenges and opportunities (Dorsman et al., 2023; Levante et al., 2024).

Recent systematic reviews (e.g., Cervellione et al., 2025; Levante et al., 2024) on TD siblings have outlined the main constructs investigated in this population. Levante et al. (2024), focusing on a broad range of disabilities, highlighted that the quality of the sibling relationship, the caregiving role of TD siblings, and their emotional and behavioural adjustment were the recurring dimensions explored. Cervellione et al. (2025), in a review specifically targeting TD siblings of individuals with autism spectrum disorder, explored domains of social and personal functioning, highlighting outcomes ranging from increased empathy and resilience to heightened anxiety, depression, and social difficulties.

Summarising, on the one hand, TD siblings may experience a range of negative outcomes, including elevated levels of anxiety, depression, social isolation, behavioural difficulties, and poor sibling relationship quality increasing the risk of both internalising and externalising psychopathologies (Giallo & Gavidia-Payne, 2006; Hastings, 2003; O’Neill & Murray, 2016; Petalas et al., 2009; Sharpe & Rossiter, 2002; Williams et al., 2010). On the other hand, a growing body of research has also documented positive outcomes, such as heightened empathy, emotional sensitivity, psychosocial competence, and a sense of fulfilment derived from their supportive role within the family (Kaminsky & Dewey, 2001; Moyson & Roeyers, 2012; Opperman & Alant, 2003; Pilowsky et al., 2004; Takataya et al., 2019).

Nevertheless, no studies have explored the reaction to the diagnosis process in the TD sibling population. Only a recent study (Lecciso et al., 2025a) addressed this process for the first time, showing that they may also undergo an emotional and cognitive process similar to that observed in parents. Specifically, the study involved parent-TD sibling dyads of individuals with disabilities and found that the TD sibling resolution significantly impacted the quality of the sibling relationship. Furthermore, the TD sibling resolution was predicted by parental resolution, supporting the hypothesis of the intergenerational transmission of internal working models (Bowlby, 1982).

Given the well-documented relevance of the reaction to the diagnosis process in parents (Lecciso et al., 2013b; Marvin & Pianta, 1996; Milshtein et al., 2010; Sher-Censor & Shahar-Lahav, 2022) and the central role of TD siblings in the present and future of the brother/sister with disability (Brolin et al., 2024; Chiu, 2022; Guidotti et al., 2021; Levante et al., 2024), exploring this process within the TD sibling population represents a novel contribution to the literature. It is not surprising that the lack of studies implies the lack of measures assessing this process in TD siblings.

The present study aimed to fill this gap by adapting for use with typically developing siblings a parent report questionnaire developed to assess how parents process their child’s diagnosis of disability. Given the novelty of the topic, the study presented the preliminary psychometric properties of the measure.

### 1.1. Measure for Assessing the Reaction to the Diagnosis Process

To date, two measures have been developed in the literature to investigate the reaction to the diagnosis process in parents: The Reaction to Diagnosis Interview (RDI; Marvin & Pianta, 1996) and the Reaction to Diagnosis Questionnaire (RDQ; Sher-Censor et al., 2019).

The RDI (Marvin & Pianta, 1996) is a semi-structured interview designed to explore the psychological process parents undergo following the diagnosis of a disability and/or chronic illness in the child. The measure allows for an in-depth examination of parents’ internal working models (Bowlby, 1982), which encompass their representations of themselves, their child, and their relationship. The coding of the interview classifies parents as resolved or unresolved regarding the diagnosis of their child. Although the RDI provides a thorough understanding of parents’ psychological experiences, its administration and coding require significant time, resources, and specialised training (Sher-Censor et al., 2019). To address the need for an easy-to-administer and user-friendly measure, the RDQ (Sher-Censor et al., 2019) has recently been developed. The authors generated 42 items inspired by the elements of resolution and lack of resolution, as defined by the Reaction to Diagnosis Interview (RDI) coding manual (Marvin & Pianta, 1996). The items encompass core aspects of resolution and lack of resolution. For instance, based on the element of resolution “*Balanced statements regarding benefits of the experience to the self”*, the item *“My child has enriched my life by being a child with special needs”* was developed. In contrast, to reflect elements of lack of resolution, such as “*Cognitive distortions concerning the permanence of the child’s diagnosis or condition*”, the item “*I believe that my child’s diagnosis is incorrect*” was developed. The representativeness of the items has been evaluated by two experts in RDI research, supporting the content validity of the measure. Lastly, a pilot study was conducted with two mothers of preschool-aged children diagnosed with cerebral palsy. The qualitative feedback collected during the pilot study was used to refine the wording of several items.

The ultimate version of the RDQ is a 42-item self-report questionnaire assessing the resolution or lack of resolution of the child’s diagnosis in parents. The response options are rated on a 5-point Likert scale (1 = *strongly disagree*; 5 = *strongly agree*; theoretical range: 1–5). The total score is calculated as the average of all items, with a higher score indicating a higher degree of resolution of the diagnosis.

Four studies have been retrieved on the application of the questionnaire. This first study (Sher-Censor et al., 2019) involved 75 Israeli mothers of children with cerebral palsy (CP; *n* = 37) or developmental delay (DD; *n* = 38). Mothers of children with CP had a mean age of 34.92 years (*SD* = 5.92), while mothers of children with DD had a mean age of 33.18 years (*SD* = 5.58). Children with CP had a mean age of 64.89 months (*SD* = 27.50), 25.34% of whom were female; children with DD had a mean age of 51.87 months (*SD* = 11.26), with 17.33% female. RDQ score and RDI classifications were not significantly associated with most socio-demographic features (e.g., child’s sex and age, maternal education level). However, both measures were significantly associated with children’s functioning profile in the DD group (RDQ: *r* = 0.58, *p* < 0.001; RDI: *χ*^2^ = 7.49, *p* = 0.006), and only the RDQ correlated with the severity of disability in the CP group (*r* = −0.34, *p* = 0.044).

In terms of internal consistency, the RDQ demonstrated good reliability, with Cronbach’s alphas of 0.89 for the CP group and 0.78 for the DD group. Regarding construct validity, mothers classified as resolved according to the RDI reported significantly higher RDQ score compared to those unresolved (*M* = 4.30, *SD* = 0.31 vs. *M* = 3.95, *SD* = 0.44; *p* < 0.001, *η*^2^ = 0.23), confirming the RDQ’s ability to discriminate between RDI classification. In addition, evidence of construct validity was provided by the Receiver Operating Characteristic analysis comparing the RDQ with the RDI. The RDQ showed adequate discriminative accuracy (area under the curve = 0.754, *p* < 0.001), a result consistent with accepted benchmarks for psychological measures (Youngstrom, 2013). Although no exploratory factor analyses were conducted, the authors conceptually proposed a unidimensional structure for the RDQ, suggesting that the measure reflects a single underlying construct, that is, the resolution of diagnosis.

In the study by Naicker et al. (2024), the RDQ was used to investigate the mediating role of different forms of hope (i.e., child-focused, parental, and social hope) on the relationship between parental resolution of diagnosis and parental stress in families with children with autism. The sample consisted of 73 parents (*M* = 43.22 years, *SD* = 7.69; 97.3% mothers), whose children had been diagnosed with autism at a mean age of 5.5 years (*SD* = 3.45) and were aged, at the time of the study, on average 11.15 years old (*SD* = 4.56; 67.1% male). As declared by the study’s authors, one RDQ item was intentionally excluded to prevent conceptual overlap with denial-based hope, and one additional item was mistakenly removed (for details, refer to Naicker et al., 2024).

In terms of internal consistency, despite this reduced version, the measure demonstrated good reliability (*McDonald’s ω* = 1.20). Regarding construct validity, the RDQ score is positively correlated with the child-focused hope (*r* = 0.59, *p* < 0.001), parental hope (*r* = 0.53, *p* < 0.001), and social hope (*r* = 0.45, *p* < 0.001). The total effect model was significant (*F* = 22.86, *p* < 0.001), explaining 50.22% of the variance. The total effect of the resolution score on parental stress was significant [*β* = −0.92, *SE* = 0.11, 95% CI (−0.73, −0.70), *p* < 0.001], and the direct effect remained significant after accounting for mediators [*β* = −0.79, *SE* = 0.15, 95% CI (−0.63, −0.49), *p* < 0.001], indicating that higher resolution was associated with lower parental stress. Among the hypothesised mediators, only parental hope significantly mediated this relationship [*β* = −0.15, SE = 0.07, 95% CI (−0.40, −0.002), *p* = 0.04] (for details, refer to Naicker et al., 2024).

In the study by Sher-Censor et al. (2024), the RDQ was administered to explore the relationship between parental resolution and their emotional availability (EA). The study involved 82 Israeli families of children with autism and severe behavioural problems. The sample included 79 mothers, 69 fathers, and their children (*M* = 116.90 months, *SD* = 30.13 months; range = 61–173 months). Both parents participated in 66 families, only the mother in 13 families, and only the father in 3 families. For mothers, the mean RDQ score was 3.77 (*SD* = 0.42; empirical range = 2.85–4.87), while for fathers, it was 3.83 (*SD* = 0.36; empirical range = 3.05–4.69).

In terms of internal consistency, the RDQ demonstrated good reliability for both mothers (*α* = 0.86) and fathers (*α* = 0.85), supporting the measure’s reliability across parents’ sex. Regarding construct validity, significant positive associations were found between maternal resolution score and EA, even after controlling for the child’s sex, autism severity, and the child’s adaptive functioning. This supports the convergent validity of the RDQ among mothers, as EA is conceptually linked to a parent’s ability to emotionally engage with their child. No significant associations were observed between paternal resolution and EA, though a positive correlation between maternal and paternal RDQ scores suggested potential reciprocal influences in parents’ mental representations. Among fathers, resolution was weakly associated with years of education (*r* = 0.24, *p* = 0.049), but no significant differences were found between mothers and fathers in RDQ scores or other key measures.

In the study by Lecciso et al. (2025a), the RDQ was used for the first time in a dyadic analysis involving parents and TD siblings. Specifically, the study tested the following: (HP1) whether TD siblings’ resolution of the diagnosis of their brother/sister with disability would be a potential predictor of the quality of the sibling relationship (in terms of closeness, conflict, jealousy, self-marginalisation, and worry); (HP2) whether parental resolution would be a potential predictor of the TD siblings’ resolution. In addition, the study explored (RQ) whether being an older versus younger TD sibling influenced resolution and sibling relationship. The sample consisted of 365 parent–sibling dyads, including parents (*M* = 51.2 years, *SD* = 6.95; range = 25–64 years; 78.4% mothers) and TD siblings (*M* = 23.2 years, *SD* = 3.60; range = 18–39 years; 53.7% female) of individuals with neurodevelopmental disorders and physical disabilities.

The measure’s internal consistency was excellent for both the parents (*α* = 0.89) and the TD sibling (*α* = 0.89) population. Regarding construct validity, TD siblings’ resolution was positively associated with closeness and negatively associated with conflict, jealousy, self-marginalisation, and worry in the sibling relationship (HP1). Parental resolution was positively associated with TD siblings’ resolution (HP2), supporting the intergenerational transmission of the internal working models. The tested model showed good fit indices (*χ*^2^ = 24.9; *df* = 5; *p* < 0.001; *CFI* = 0.97; *RMSEA* = 0.10). Finally, concerning the research question (RQ), younger TD siblings reported a significantly higher resolution score than older ones. The novelty of the latter result suggested the need for further investigations.

In conclusion, although the RDQ is a relatively novel measure and only one of the four studies that administered it evaluated its psychometric properties, all the available studies provided meaningful insights into how families, particularly parents, process a child’s diagnosis. Across the studies, resolution of diagnosis consistently emerged as a theoretically and empirically relevant construct, showing associations with related psychological dimensions such as parental stress, hope, and emotional availability. Notably, only one study explored the topic in the TD siblings with promising results, underscoring the importance of adopting a whole-family perspective when addressing the psychological impact of growing a child with disability and/or chronic illness. While further validation studies are needed, the RDQ emerges as a promising, theory-driven, and user-friendly measure for assessing the resolution of diagnosis in both parents and TD siblings.

### 1.2. Aims of the Study

The present study aimed to explore the preliminary psychometric properties of the Reaction to Diagnosis Questionnaire—Sibling Version (RDQ-S), an adaptation of the original parent report version (Sher-Censor et al., 2019). In particular, the study investigates the internal consistency of the measure, as an indicator of its reliability, and explores its structure through exploratory factor analysis.

## 2. Materials and Methods

### 2.1. Back-Translation and Adaptation of the RDQ-S

To ensure the linguistic and cultural appropriateness of the measure for use in the Italian context, a rigorous translation and cross-cultural adaptation process was carried out. This process followed well-established and internationally recognised procedures for forward and backwards translation (Brislin, 1970) and was guided by protocols for adapting measures across cultures (Beaton et al., 2000). The primary aim was to retain the conceptual equivalence of the original items and minimise semantic discrepancies that may arise during translation (Regmi et al., 2010).

The original English version was independently translated into Italian by two bilingual individuals fluent in both English and Italian, following the principle of independent translation (Gudmundsson, 2009). Translators were explicitly instructed to avoid literal translation and instead focus on conveying the intended meaning. This phase resulted in two independent Italian versions of the questionnaire. These versions were then reviewed by members of the research team, who compared and discussed discrepancies to reach conceptual equivalence and produce a single Italian version.

Subsequently, the Italian version was back-translated into English by two bilingual professionals who were not exposed to the original version. This step allowed for verification of item accuracy and the conceptual fidelity of the translation. The resulting English version was then compared with the original to ensure that the meaning of the items had been preserved.

Following the back-translation phase, the questionnaire was further adapted from the parental version to a TD sibling-specific version. This adaptation involved replacing references to the “*child*” with references to the “*brother/sister*” with disability. For example, the item “*Today I can see my child’s difficulties as well as his/her strengths and achievements*” was adapted to “*Today I can see my brother/sister’s difficulties as well as his/her strengths and achievements*”.

### 2.2. Measures

*TD Sibling Resolution of the Diagnosis.* The Reaction to Diagnosis—Sibling Version (RDQ-S) assesses the TD sibling’s resolution of the diagnosis of the brother/sister. The response rate varied on a 5-point Likert scale (1 = *strongly disagree*; 5 = *strongly agree*). The resolution score is calculated as the average of all items, with a higher score indicating a higher degree of resolution of the diagnosis. The items included in the adapted version of the questionnaire (RDQ-S) are provided in the Supplementary Material (in both English and Italian) [*M* = 3.87, *SD* = 0.44; *α* = 0.90].

*Severity of Disability.* The Barthel Index (Mahoney & Barthel, 1965) measures the severity of disability of the brother/sister. This index evaluates the need for assistance in activities of daily living (e.g., eating, dressing, and personal hygiene). Response options are rated on a 5-point Likert scale (1 = *completely independent*; 5 = *unable to perform*). The total score is calculated by summing all items, with a higher score indicating greater severity of disability [*M* = 3.71, *SD* = 1.24; *α* = 0.94].

### 2.3. Study Design

The cross-sectional study was conducted in Italy between April and September 2024 through an online survey hosted on LimeSurvey. The research was approved by the University Ethical Committee for Research in Psychology at the Department of Human and Social Sciences of the University of Salento, Italy (Approval No. 92949). Participants were recruited through convenience sampling by disseminating the survey link via social media platforms, associations for families of individuals with disabilities, and university networks. To encourage complete responses and minimise missing data, each item was mandatory. However, as stated in the informed consent form, participation was entirely voluntary, and participants were free to withdraw at any point, ensuring compliance with ethical research standards. No payment or any compensation was provided to participate.

The inclusion criteria for the study were as follows: (1) age between 14 and 40 years, (2) having a brother/sister with neurodevelopmental disorders (NDDs) or physical disabilities (PDs); (3) having no disabilities, and (4) fluency in Italian. Participants provided informed consent by electronically agreeing to the e-consent form before participating. For under-18 participants, informed consents were provided by parents. A total of 1151 initial accesses to the survey link were recorded. Of these, 623 participants completed the questionnaire, resulting in a response rate of approximately 54%. Among the remaining participants (*n* = 528), 392 did not complete the questionnaire, while 136 were excluded, as they did not meet the inclusion criteria.

The survey consists of two main sections. The first one aimed to collect socio-demographic features concerning both the TD sibling and their brother/sister with disability. For the TD sibling, information included age, sex, marital status, educational level, and sibship (being the older TD sibling vs. the younger TD sibling). For the brother/sister with disability, participants were asked to provide information on age, sex, marital status, educational level, and the type of disability (NDDs vs. PDs). The second section aimed to investigate the TD siblings’ resolution of the diagnosis concerning their brother/sister’s disability. In addition, the severity of the brother/sister’s disability was assessed.

### 2.4. Participants

In total, 623 completed the e-survey (TD siblings: *M* = 24.6 years, *SD* = 5.76, age range = 14–40 years; females = 58.6%). A total of 259 TD siblings were from families of individuals with NDDs (*M* = 20.9 years, *SD* = 7.76, age range = 4–64 years; females = 28.57%), and 364 TD siblings were from families of individuals with PDs (*M* = 24.5 years, *SD* = 8.93, age range = 2–60 years; females = 48.18%).

Table 1 presents the socio-demographic features of TD siblings and their brothers/sisters with disability.

### 2.5. Statistical Plan

Analyses were conducted using Jamovi v. 2.6.26 (The Jamovi Project, 2024). Due to the mandatory response option, no imputation techniques for missing data were required. Power analysis for group comparisons was performed using the JPower package in Jamovi. The normality of data distribution was assessed through the Shapiro–Wilk test, and the homogeneity of variances was tested using Levene’s test. Group comparisons were conducted to examine differences across TD sibling sex, type of disability (TD siblings of individuals with NDDs vs. those with PDs), and sibship (being the older vs. younger TD siblings). Partial correlations were computed to examine associations between the resolution score and socio-demographic features, controlling for TD sibling sex, sibship, and the type of disability of the brother/sister. Lastly, an exploratory factor analysis (EFA) was conducted to explore the structural validity of the questionnaire.

All the analyses were initially performed based on the original version of the measure, which assumes a unidimensional structure, and were subsequently replicated on the refined structure resulting from the EFA. Assumptions of factorability were assessed. The Kaiser–Meyer–Olkin (KMO) measure was used to evaluate sampling adequacy, with reference thresholds indicating moderate adequacy for values between 0.70 and 0.80, good adequacy between 0.80 and 0.90, and excellent adequacy above 0.90 (Morgan et al., 2004). Bartlett’s Test of Sphericity was also computed to determine whether the items were sufficiently intercorrelated. The selection of the optimal number of the factor(s) was based on the scree plot (eigenvalues > 1; *χ*^2^(738) = 1982; *p* < 0.001, RMSEA = 0.05 (90% CI: 0.049–0.054), which showed that the percentage of the variance decrease whether three factors were fixed (6.35%), while selecting two factors the variance was 13.39%. Thus, according to this preliminary analysis and the theoretical model (Marvin and Pianta, 1996) hypothesising two outcomes of the reaction to the diagnosis process (resolution vs. lack of resolution), the EFA was computed fixing two factors. The significance level was set at *p* < 0.050.

## 3. Results

### 3.1. Preliminary and Descriptive Analyses

The power analysis for group comparisons revealed that a minimum sample of 172 is adequate for drawing valid conclusions regarding significant effects, ensuring the robustness and the interpretations of results (*δ* ≥ 0.5 with a probability of at least 0.90).

**Figure 1:**
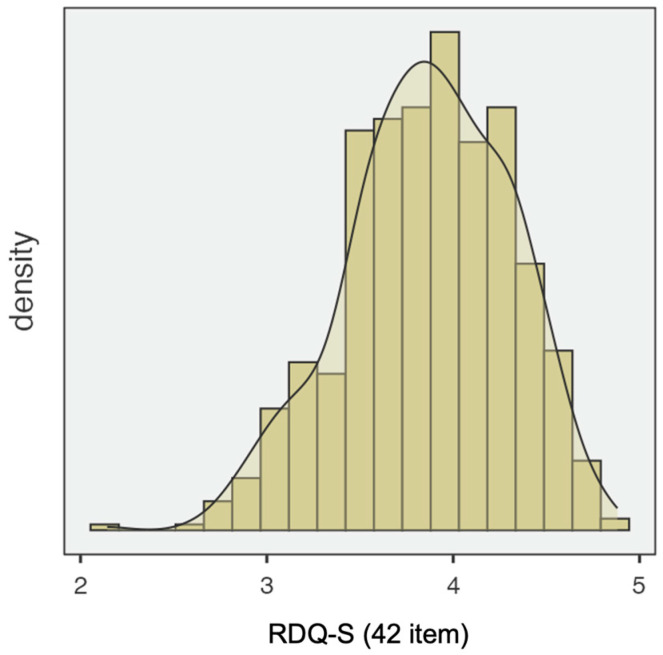
Distribution of the RDQ-S total score.

Shapiro–Wilk test indicated non-normality of the data (skewness = −0.331; Kurtosis = −0.106; see Figure 1).

Levene’s test showed no significant differences in variances across groups, supporting homogeneity of variance.

Mann–Whitney U tests according to TD siblings’ sex, type of disability (TD siblings of individuals with NDDs vs. TD siblings of individuals with PDs), and sibship (being the older TD sibling vs. the younger TD sibling) showed no differences between groups on the TD sibling resolution score. Table 2 shows the results of the group comparisons.

Spearman’s rho partial correlations revealed a negative association between the TD sibling resolution score and the severity of the disability of the brother/sister (*r* = 0.74, *p* < 0.001).

Regarding the internal consistency of the measure [*M* = 3.86, *SD* = 0.44], the analysis yielded *Cronbach’s α* = 0.90 and *McDonald’s ω* = 0.91, indicating excellent reliability. These reliability indices were obtained by applying the original unidimensional structure developed for the parental version of the measure to the adapted TD sibling version. However, since the original RDQ for parents had not undergone exploratory factor analysis to empirically verify its structure, we extended the validation process by conducting an exploratory factor analysis on the TD sibling version.

### 3.2. Exploratory Factor Analysis

The Kaiser–Meyer–Olkin (KMO) measure indicated excellent sampling adequacy (KMO = 0.93). Bartlett’s Test of Sphericity was also significant [*χ*^2^(861) = 10.680, *p* < 0.001], confirming the suitability of the data for factor analysis. The factor loadings of the retained items are presented in Table 3.

Items with loadings below 0.30 (i.e., items 41, 35, and 2) were excluded. Additionally, we removed items that substantially cross-loaded on both factors (i.e., items 25, 10, and 20), as well as items that, although statistically adequate, were conceptually inconsistent with the thematic content of their assigned factor (i.e., items 36 and 18). Item 12 was also removed because, despite referencing unresolved representations, it loaded onto the factor reflecting resolution. Items were removed iteratively, with the factor structure re-evaluated after each step. The final structure included 32 items loading onto two distinct factors. The first factor reflected the lack of resolution (e.g., “*I am still upset regarding the way my brother/sister was diagnosed*”), while the second captured resolution (e.g., “*I feel that my brother/sister’s condition is improving*”). Given the complexity of the model and its theoretical alignment, this two-factor solution represents the best-fitting and most interpretable configuration. However, these results should be considered preliminary indicators of model fit and require confirmation in future studies with independent samples.

**Figure 2:**
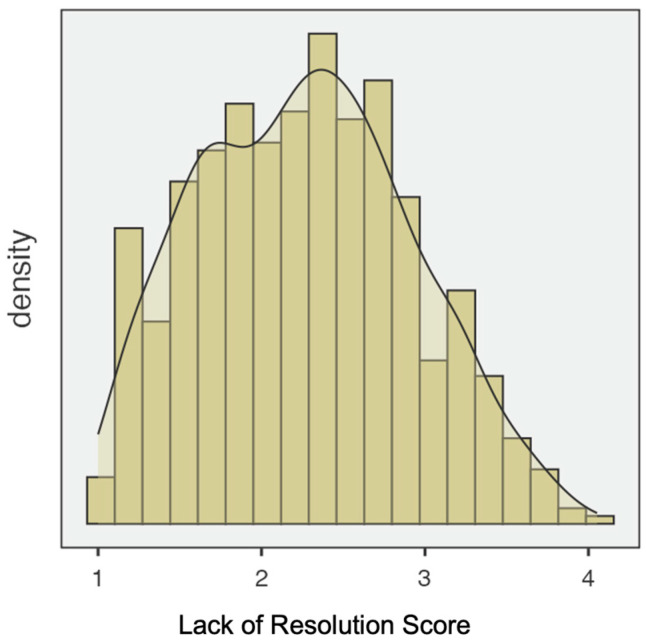
Distribution of the lack of resolution score (RDQ-S; 32 items).

**Figure 3:**
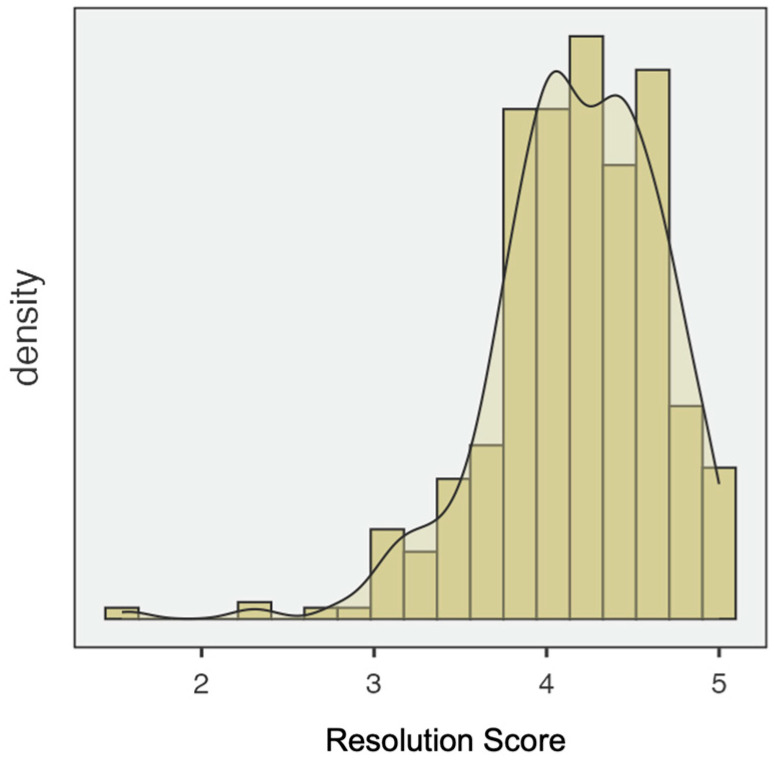
Distribution of the resolution score (RDQ-S; 32 items).

Following the novel’s two-factor structure (lack of resolution and resolution), descriptive analyses were conducted for each factor. For the first one, lack of resolution, Shapiro–Wilk test indicated that the distribution deviated from normality (skewness = 0.176; Kurtosis = −0.654) (see Figure 2). For the second factor, resolution, the Shapiro–Wilk test also showed non-normality of the data, with skewness = −1.00 and kurtosis = 2.59 (See Figure 3).

For both factors, Levene’s test indicated the variances are equal across the groups, supporting the assumption of homogeneity of variance. Mann–Whitney U tests conducted according to TD sibling sex, type of disability, and sibship revealed no significant differences between groups on either the lack of resolution or the resolution factor. The results of the group comparisons are reported in Table 4.

Spearman’s rho partial correlations revealed that the age of the TD sibling is negatively associated with resolution (*r* = −0.122, *p* ≤ 0.01), suggesting that older TD siblings show lower levels of resolution. Similarly, the age of the brother/sister with disability is negatively correlated with resolution *(r* = −0.130, *p* ≤ 0.01). The severity of the disability is positively associated with lack of resolution (*r* = 0.311, *p* ≤ 0.001) and negatively with resolution (*r* = −0.306, *p* ≤ 0.001), indicating that greater disability severity is linked to a more unresolved representation in the TD sibling.

Regarding the internal consistency of the scales, both factors demonstrated strong internal reliability, in line with the criteria proposed by Fornell and Larcker (1981). Further details are reported in Table 5.

## 4. Discussion

The study explored the preliminary psychometric properties of the Reaction to Diagnosis Questionnaire—Sibling Version (RDQ-S). The questionnaire has been adapted for the TD sibling population from the original version developed for parents of a child with a disability. The adaptation and validation of this measure mark an important step in understanding how TD siblings process the diagnosis of disability of their brother/sister. To our knowledge, the research field was under-investigated in both research and clinical practice. Evidence has extensively documented the reaction to the diagnosis process in parents (Lecciso et al., 2013a; Marvin & Pianta, 1996; Oppenheim et al., 2009; Shah et al., 2011), emphasising positive cascade effects on the attachment bond and the overall family functioning by the resolution of the diagnosis. Little is known about how TD siblings, who often play a central role in the brother/sister’s present and future, experience and integrate the diagnosis across their lifespan.

After following a back-translation and linguistic adaptation process, the current cross-sectional study on TD siblings aimed at examining the reliability and exploring the structural validity of the RDQ-S. A total of 623 TD siblings of individuals with NDDs (*n* = 259) and physical disability (*n* = 364) participated in the study.

Preliminary analyses focused on the unidimensional 42-item structure derived from the parental version. Group comparisons did not reveal significant differences in resolution score based on TD sibling sex, type of disability of the brother/sister, or TD sibling sibship. This means that there was a good degree of consistency in how TD siblings process the diagnosis across these socio-demographic features. When controlling for these socio-demographic features, partial correlations highlighted a significant negative association between resolution and the severity of the brother/sister’s disability. The results suggested that the more severe the disability was, the greater the emotional difficulty in processing the diagnosis was reported by the TD sibling. The reasons why the TD siblings may be related to different daily life issues, for instance, the increased caregiving demands or more visible disruptions to family life. Thus, special attention could be devoted to the TD sibling experience when extreme disability occurs in the family. The 42-item measure showed excellent internal consistency, with Cronbach’s *α* and McDonald’s *ω* exceeding 0.90, supporting the measure’s reliability.

Although a unidimensional structure has been hypothesised by the original authors for the RDQ (Sher-Censor et al., 2019), no factor analysis has explored the factor structure of the measure. The current study preliminarily explored the questionnaire structure via an EFA. Based on the percentage of the variance which increased when two factors were considered and the theoretical model (Marvin & Pianta, 1996) hypothesising two outcomes of the reaction to the diagnosis process (resolution vs. lack of resolution), a two-factor structure was run.

The sample met all statistical assumptions for factor analysis, and the iterative removal of items with low loadings, cross-loadings, or conceptual inconsistencies resulted in the refined 32-item version based on the two factors.

Similarly to parents, the lack of resolution factor captures TD siblings’ ongoing emotional distress, denial, confusion, or preoccupation related to the brother/sister’s condition. It encompasses difficulties in making meaning of the diagnosis, persistent ambivalence, and an inability to integrate the experience into one’s broader life narrative. Conversely, the resolution factor reflects an emotionally integrated and accepting representation of the brother/sister’s disability. It includes items that capture the ability to make sense of the diagnosis, to maintain a balanced view of the brother/sister, and to engage in future-oriented thinking without being overwhelmed by negative emotions. Given the exploratory nature of the study, these results require further investigations testing CFA in future research using larger and more diverse samples.

Regarding the preliminary and descriptive analyses on the refined 32-item version, the results revealed that no significant group differences emerged based on TD sibling sex, type of disability of the brother/sister, or TD sibling sibship, in line with the results from the original 42-item version.

The absence of sex differences is particularly noteworthy. Although the resolution has not previously been examined in TD siblings, prior research on this population has shown that TD sisters are more likely to assume caregiving roles and report higher levels of negative emotions towards their brother/sister with disability compared to TD brothers (Levante et al., 2024; O’Neill & Murray, 2016; Yaldiz et al., 2021). As a sequel, it would be expected to see a sex difference in terms of a greater likelihood of a lack of resolution in female TD siblings. However, the female-prevalent sample size recruited in the existing evidence (Braconnier et al., 2018; Chiu, 2022; Levante et al., 2023; Travers et al., 2020) leads to reading the existing results carefully. In the current study, the more balanced sample may have allowed for a more complete representation of TD brothers, potentially accounting for this unexpected result. Nevertheless, given the novelty of this area, further investigation is warranted.

As for the type of disability of the brother/sister, our results align with previous research on parental resolution, which suggested that it is not the type of diagnosis but rather its severity that influences the reaction to the diagnosis process (Hutman et al., 2009; Lecciso et al., 2013a; Marvin & Pianta, 1996; Pianta et al., 1999; Poslawsky et al., 2014; Sher-Censor & Shahar-Lahav, 2022). A similar pattern may, therefore, be hypothesised for TD siblings as well.

Sibship remains generally underexplored in the broader literature on TD siblings. One possible explanation for the absence of significant differences is that the reaction to the diagnosis process may not be shaped by birth order alone but rather by a complex interplay of factors, such as family dynamics and parental expectations. Moreover, both older and younger siblings may face distinct but comparable challenges. For instance, older TD siblings may perceive greater responsibility, whereas younger TD siblings may experience confusion or emotional distance. These differences could potentially balance each other out in terms of resolution and lack of resolution. As this is an unexplored area, further research is needed.

Correlational analyses revealed that older TD siblings reported a low level of resolution; in addition, the older the brother/sister with disability, the lower the resolution. This pattern may reflect the cumulative psychological impact of growing up in a family system long shaped by disability-related challenges. Over time, TD siblings may internalise relational dynamics, emotional burdens, or caregiving roles in ways that become increasingly stable and difficult to revise. In this sense, it can be hypothesised that prolonged exposure may limit the flexibility needed to develop a more coherent and accepted representation of the brother/sister’s disability, thus reducing the likelihood of achieving resolution.

Furthermore, greater severity of the brother/sister’s disability was associated with both lower resolution and higher lack of resolution. This suggests that the more challenging the condition, the harder it may be for TD siblings to construct a coherent and adaptive narrative around the diagnosis, emphasising how severity, not merely diagnosis type, plays a crucial role in shaping their reaction to the diagnosis process.

The results showed an excellent internal reliability of the two factors. These results supported the RDQ-S as a promising measure in capturing how TD siblings come to terms with the diagnosis of disability within their family context.

Taken together, the results of this study underscore the relevance of investigating how TD siblings process the diagnosis of their brother/sister, not only as a matter of research interest but also as a potential area for psychological support and intervention. Understanding TD siblings’ psychological experience can inform family-centred practices and support professionals in identifying TD siblings who may be at risk of a lack of resolution, thereby helping to prevent the psychopathological outcomes already documented in the literature (Williams et al., 2010; O’Neill & Murray, 2016; Rossetti & Hall, 2015).

Although exploratory and preliminary, these results are encouraging and suggest that the RDQ-S is a promising measure. The scale’s theoretical coherence and preliminary psychometric validity support its potential as a valuable resource in both research and practice. However, confirmatory factor analysis is essential for validating the proposed structure and providing a more robust assessment of its factorial validity. These results lay the groundwork for more extensive validation efforts and highlight the importance of further longitudinal and cross-cultural studies. Moreover, the RDQ-S could inform the development of targeted intervention programs aimed at supporting the psychological adjustment of TD siblings, as they navigate the complex emotional and relational dynamics associated with disability within the family system.

## 5. Strengths, Limitations, and Future Directions

The primary strengths of the study lie in its preliminary exploration of the previously unexplored reaction to the diagnosis process within the TD sibling population. The measure is grounded in Bowlby’s attachment theory (Bowlby, 1982) and Marvin and Pianta’s frameworks (Marvin & Pianta, 1996), which provide a strong theoretical foundation.

A further strength is the inclusion of an exploratory factor analysis, which was not conducted in the original version of the instrument developed for parents. Moreover, the sample included TD siblings of individuals with two types of disabilities (NDDs and PDs), which differ markedly in terms of onset, manifestation, and impact on daily functioning. NDDs, such as autism spectrum disorder and intellectual disabilities, typically involve pervasive impairments in cognitive, social, and communicative domains (Backer van Ommeren et al., 2017; Camargo et al., 2014; Levante et al., 2020). PDs, including conditions like cerebral palsy or muscular dystrophy, primarily affect motor and physical functioning and may be more visible, though not necessarily associated with cognitive impairment (Auyeung et al., 2008; Church et al., 2025). Including both types of disability allowed for a broader understanding of how different disabilities may shape the resolution process in TD siblings.

However, the study has some limitations that should be taken into account. First, it is a preliminary investigation based on a cross-sectional design and a culturally homogeneous sample, which limits the generalisability of the results. Additionally, this study did not collect information regarding the specific diagnosis of the brother/sister. Furthermore, the survey required participants to respond to all questions. This methodological choice was intended to minimise missing data, ensuring the completeness of all items. While we cannot determine the exact reasons for withdrawal, we acknowledge that allowing participants to skip individual questions might have better accommodated their preferences and potentially improved completion rates. This may be a valuable consideration for future studies employing similar methodologies.

The results of this study open up several important avenues for future research. Future research should aim to confirm the factor structure using confirmatory factor analysis and further explore the scale’s psychometric properties in more diverse samples. Additionally, it will be crucial to examine the predictive capacity of the RDQ-S concerning psychological, relational, and adaptive outcomes among TD siblings. Longitudinal studies using the RDQ-S could provide valuable insights into the evolving representation of disability throughout the lifespan.

From a clinical perspective, the RDQ-S holds potential as a valuable tool in health care services for identifying TD siblings who struggle with emotionally and cognitively processing their brother/sister’s diagnosis. This could facilitate the implementation of targeted, family-centred intervention programs that address the often-overlooked needs of TD siblings within psychological support pathways. Crucially, these findings underscore the importance of adopting a systemic approach when a diagnosis occurs within a family, one that considers not only the experiences of parents (Milshtein et al., 2010; Sher-Censor & Shahar-Lahav, 2022) and individuals with disability (De Carlo et al., 2024; Martis et al., 2024) but also those of TD siblings. In this context, the present study represents an important first step toward fostering a more integrated understanding of the family dynamics associated with disability.

## Figures and Tables

**Table 1:** Socio-demographic information.

Educational Level	
TD siblings of individuals with NDD	Low (up to 8 years of education) = 13% Intermediate (up to 13 years of education) = 60% High (13 or more years of education) = 27%
Individuals with NDD	No formal education = 15% Low (up to 8 years of education) = 32% Intermediate (up to 13 years of education) = 52% High (13 or more years of education) = 1%
TD siblings of individuals with physical disability	Low (up to 8 years of education) = 12% Intermediate (up to 13 years of education) = 66% High (13 or more years of education) = 22%
Individuals with physical disability	No formal education = 8% Low (up to 8 years of education) = 30% Intermediate (up to 13 years of education) = 56% High (13 or more years of education) = 6%
Marital status	
TD siblings of individuals with NDD	With a partner = 13.50% Without a partner = 86.50%
Individuals with NDD	With a partner = 5% Without a partner = 95%
TD siblings of individuals with physical disability	With a partner = 23% Without a partner = 77%
Individuals with physical disability	With a partner = 9% Without a partner = 91%
Employment status	
TD siblings of individuals with NDD	Employed = 41% Unemployed = 59%
Individuals with NDD	Employed = 10% Unemployed = 90%
TD siblings of individuals with physical disability	Employed = 55% Unemployed = 45%
Individuals with physical disability	Employed = 18% Unemployed = 82%
Sibship	
Older vs. younger TD siblings of individuals with NDD	Older TD sibling = 61% Younger TD sibling = 39%
Older vs. younger TD siblings of individuals with physical disability	Older TD sibling = 58% Younger TD sibling = 42%

**Table 2:** Mann–Whitney *U*-tests (42 item).

	TD Sibling Sex			Brother/Sister Type of Disability			TD Sibling Sibship		
	TD Sisters *M* (*SD*)	TD Brothers *M* (*SD*)	*U*; *p*	NDDs *M* (*SD*)	PDs *M* (*SD*)	*U*; *p*	Older *M* (*SD*)	Younger *M* (*SD*)	*U*; *p*
TD Sibling Resolution Score	3.86 (0.43)	3.86 (0.45)	1.654; 0.78	3.87 (0.41)	3.85 (0.46)	4.650; 0.77	3.85 (0.45)	3.89 (0.42)	4.475 (0.31)

**Table 3:** Factor loadings of the items resulting from the EFA with the maximum likelihood extraction method combined with an oblimin rotation. Only loadings greater than 0.30 are displayed.

	FACTOR	
	Lack of Resolution	Resolution
28. It is difficult for me to stop thinking about my brother/sister’s diagnosis and difficulties. (r)	0.766	
26. I am angry about everything that happened to my brother/sister and me. (r)	0.734	
30. I keep asking myself why this happened to me. (r)	0.732	
5. I am still upset regarding the way my brother/sister was diagnosed. (r)	0.727	
22. There isn’t a day in which I don’t think about the amount and type of treatments and interventions my brother/sister receives.	0.670	
19. Whenever I think about my brother/sister, I feel despair or sadness. (r)	0.662	
4. I often think why my brother/sister has this diagnosis. (r)	0.640	
14. Since I am aware of my brother/sister’s diagnosis, it is difficult for me to function in day-to-day life. (r)	0.630	
15. I am preoccupied with searching for reasons for my brother/sister’s difficulties. (r)	0.623	
31. I am very concerned about my brother/sister’s future. (r)	0.581	
32. Since I found out about my brother/sister’s diagnosis, I feel powerless and there is no joy in my life. (r)	0.562	
13. I am preoccupied with thinking and asking what I did wrong so that this has happened to me, that I have a brother/sister with special needs. (r)	0.549	
23. I feel very confused about my brother/sister’s diagnosis. (r)	0.511	
9. When I think about having a brother/sister with special needs, I feel guilty. (r)	0.509	
37. I continue to take my brother/sister to receive additional medical opinions about her/his diagnosis. (r)	0.491	
25. I remember every detail of the moment I found out my brother/sister’s diagnosis as if it were yesterday. (r)	0.474	0.350
10. It is difficult for me to treat my brother/sister as a “typical” person. (r)	0.394	−0.351
6. I want to treat my brother/sister like any other brother/sister, but I am not successful in doing so. (r)	0.378	
24. I feel that my brother/sister suffers because of me. (r)	0.375	
34. I am still angry regarding the way my brother/sister’s diagnosis was given to me. (r)	0.309	
21. I believe that the diagnosis my brother/sister received is incorrect. (r)	0.303	
41. I think that I or someone in my family could have prevented the development of my brother/sister’s condition. (r)		
35. My brother/sister’s diagnosis did not change my life or the life of my family. (r)		
39. Today I can see my brother/sister’s difficulties as well as her/his strengths and achievements.		0.713
40. My brother/sister has characteristics and abilities that I love very much.		0.683
7. In spite of the difficulties, I see that my brother/sister is successful in facing his/her challenges.		0.673
29. When I think about my brother/sister’s future, I believe his/her life will be happy.		0.622
27. My brother/sister has enriched my life by being a brother/sister with special needs.		0.616
17. I believe that my family and I can cope with my brother/sister’s difficulties and help him/her.		0.598
16. I see that the treatments and interventions help my brother/sister.		0.533
33. My brother/sister is in an educational setting that is appropriate to his/her needs and abilities.		0.524
3. When I plan the interventions my brother/sister will receive, the most important thing for me is that he/she will be happy.		0.514
8. I feel that my brother/sister’s condition is improving.		0.512
38. I hope that my brother/sister’s condition will improve with time.		0.454
12. I am confident that my brother/sister would soon close the gap and be like any other typically developing person. (r)		0.453
36. I do not enjoy playing/spending time with my brother/sister. (r)		−0.426
20. I feel that I lost the brother/sister I hoped to have. (r)	0.377	−0.416
1. I shared my brother/sister’s diagnosis with my extended family.		0.405
18. I don’t believe that my brother/sister’s level of independence will improve in the future. (r)		−0.361
42. I feel that my feelings regarding my brother/sister’s diagnosis have changed since I found out about my brother/sister’s diagnosis.		0.361
11. I am very dissatisfied with the treatment my brother/sister is receiving. (r)		
2. I believe that my brother/sister’s diagnosis is incorrect. (r)		

Note: (r) reverse coded.

**Table 4:** Mann–Whitney *U*-tests (32 item).

	TD Sibling Sex			Brother/Sister Type of Disability			TD Sibling Sibship		
	TD Sisters *M* (*SD*)	TD Brothers *M* (*SD*)	*U*; *p*	NDDs *M* (*SD*)	PDs *M* (*SD*)	*U*; *p*	Older *M* (*SD*)	Younger *M* (*SD*)	*U*; *p*
Lack of Resolution	2.27 (0.64)	2.25 (0.66)	4.5570; 0.47	2.25 (0.61)	2.27 (0.67)	4.6997; 0.094	2.29 (0.66)	2.22 (0.063)	4.4341; 0.23
Resolution	4.18 (0.49)	4.15 (0.49)	4.5721; 0.52	4.19 (0.48)	4.15 (0.49)	4.5554; 0.47	4.16 (0.51)	4.17 (0.45)	4.6667; 0.88

**Table 5:** Descriptive statistics and internal consistency for each factor.

Factor	*M* (*SD*)	*Cronbach’s α*	*McDonald’s* *ω*
Lack of Resolution	2.26 (0.65)	0.911	0.914
Resolution	4.17 (0.49)	0.846	0.856

## Data Availability

The authors will share the dataset upon request.

## Supplementary Materials

The following supporting information can be downloaded at https://www.mdpi.com/article/10.3390/ejihpe15080147/s1, Table S1: RDQ-S (32-item English Version), Table S2: RDQ-S (32-item Italian Version).
