# Supplementary material for: The Reaction to Diagnosis Questionnaire—Sibling Version: A Preliminary Study on the Psychometric Properties
Source: Eur J Investig Health Psychol Educ. 2025 Jul 29;15(8):147. doi: 10.3390/ejihpe15080147 (PMC12385898; doi:10.3390/ejihpe15080147)
Supplement: Supplementary file 1 [file ejihpe-15-00147-s001.zip › ejihpe-3698125-supplementary.pdf]

Supplementary Material

Table S1. RDQ-S (32-item English Version)

	Strongly Disagree (1)	Disagree (2)	Neutral (3)	Agree (4)	Strongly Agree (5)
1. I shared my brother/sister's diagnosis with my extended family.					
3. When I plan the interventions my brother/sister will receive, the most important thing for me is that he/she will be happy.					
4. I often think why my brother/sister has this diagnosis.					
5. I am still upset regarding the way my brother/sister was diagnosed.					
6. I want to treat my brother/sister like any other brother/sister, but I am not successful in doing so.					
7. In spite of the difficulties, I see that my brother/sister is successful in facing his/her challenges.					
8. I feel that my brother/sister's condition is improving.					
9. When I think about having a brother/sister with special needs, I feel guilty.					
13. I am preoccupied with thinking and asking what I did wrong so that this has happened to me, that I have a brother/sister with special needs.					
14. Since I am aware of my brother/sister's diagnosis, it is difficult for me to function in day-to-day life.					
15. I am preoccupied with searching for reasons for my brother/sister's difficulties.					
16. I see that the treatments and interventions help my brother/sister.					
17. I believe that my family and I can cope with my brother/sister's difficulties and help him/her.					
19. Whenever I think about my brother/sister, I feel despair or sadness.					
21. I believe that the diagnosis my brother/sister received is incorrect.					
22. There isn't a day in which I don't think about the amount and type of treatments and interventions my brother/sister receives.					
23. I feel very confused about my brother/sister's diagnosis.					
24. I feel that my brother/sister suffers because of me.					
26. I am angry about everything that happened to my brother/sister and me.					
27. My brother/sister has enriched my life by being a brother/sister with special needs.					

28. It is difficult for me to stop thinking about my brother/sister's diagnosis and difficulties.					
29. When I think about my brother/sister's future, I believe his/her life will be happy.					
30. I keep asking myself why this happened to me.					
31. I am very concerned about my brother/sister's future.					
32. Since I found out about my brother/sister's diagnosis, I feel powerless and there is no joy in my life.					
33. My brother/sister is in an educational setting that is appropriate to his/her needs and abilities.					
34. I am still angry regarding the way my brother/sister's diagnosis was given to me.					
37. I continue to take my brother/sister to receive additional medical opinions about her/his diagnosis.					
38. I hope that my brother/sister's condition will improve with time.					
39. Today I can see my brother/sister's difficulties as well as her/his strengths and achievements.					
40. My brother/sister has characteristics and abilities that I love very much.					
	My feelings have changed a lot for the worse (1)	(2)	(3)	(4)	My feelings have changed positively a lot (5)
42. I feel that my feelings regarding my brother/sister's diagnosis have changed since I found out about my brother/sister's diagnosis.					

Table S2. RDQ-S (32-item Italian Version)

	Fortemente in Disaccordo	Disaccordo	Nè in disaccordo, nè d'accordo	D'accordo	Fortemente D'accordo
1. Condivido la diagnosi di mio/a fratello/sorella con la mia famiglia estesa.					
3. Quando mi capita di pianificare interventi/terapie che mio/a fratello/sorella riceverà, la cosa più importante per me è che lui/lei sia felice					
4. Spesso penso al motivo per cui perché mio/a fratello/sorella abbia questa diagnosi.					
5. Sono ancora turbato/a per la diagnosi a mio/a fratello/sorella.					
6. Voglio trattare mio/a fratello/sorella come qualsiasi altro fratello/sorella, ma non riesco a farlo.					
7. Nonostante le difficoltà, vedo che mio/a fratello/sorella affronta con successo le sue sfide.					
8. Sento che la condizione di mio/a fratello/sorella sta migliorando.					
9. Mi sento in colpa quando penso di avere un/a fratello/sorella con diagnosi.					
13. Penso e mi chiedo cosa ho fatto di male perché mi sia successo questo, ossia perché ho un/a fratello/sorella con diagnosi.					
14. Da quando sono consapevole della diagnosi di mio/a fratello/sorella, è difficile per me affrontare la vita quotidiana.					
15. Sono impegnato/a a ricercare le ragioni delle difficoltà di mio/a fratello/sorella.					
16. Vedo che gli interventi e le terapie aiutano mio/a fratello/sorella.					
17. Credo che la mia famiglia e io possiamo fronteggiare le difficoltà di mio/a fratello/sorella e aiutarlo/a.					
19. Ogni volta che penso a mio/a fratello/sorella, mi sento disperato/a e triste.					
21. Credo che la diagnosi che mio/a fratello/sorella ha ricevuto sia non corretta.					
22. Non c'è giorno in cui non pensi alla quantità e al tipo di interventi e terapie che mio/a fratello/sorella riceve.					
23. Mi sento molto confuso/a riguardo alla diagnosi di mio/a fratello/sorella.					
24. Sento che mio/a fratello/sorella soffre a causa mia.					
26. Sono arrabbiato/a per tutto quello che è successo a me e a mio/a fratello/sorella.					

27. Mio/a fratello/sorella ha arricchito la mia vita, pur essendo una persona con diagnosi	
28. È difficile per me smettere di pensare alla diagnosi e alle difficoltà di mio/a fratello/sorella	
29. Quando penso al futuro di mio/a fratello/sorella, credo che la sua vita sarà anche felice.	
30. Continuo a chiedermi perché questo è successo a me e alla mia famiglia.	
31. Sono molto preoccupato/a rispetto il futuro di mio/a fratello/sorella.	
32. Da quando ho saputo della diagnosi di mio/a fratello/sorella, mi sento impotente e non c'è gioia nella mia vita.	
33. Mio/a fratello/sorella è in un contesto educativo adeguato ai suoi bisogni e alle sue capacità.	
34. Sono ancora arrabbiato/a per il modo in cui la diagnosi di mio/a fratello/sorella mi è stata comunicata.	
37. Continuo a richiedere pareri medici in merito alla diagnosi di mio/a fratello/sorella.	
38. Spero che le condizioni di mio/a fratello/sorella miglioreranno con il tempo.	
39. Oggi posso vedere le difficoltà di mio/a fratello/sorella così come i suoi punti di forza e successi.	
40. Mio/a fratello/sorella ha caratteristiche e abilità che amo molto.	
42. Sento che i miei sentimenti rispetto alla diagnosi di mio/a fratello/sorella sono cambiati positivamente nel tempo da quando l'ho saputo.	

Scoring
Compute the mean score of the following scales.

- Lack of Resolution:
  Items 28, 26, 30, 5, 22, 19, 4, 14, 15, 31, 32, 13, 9, 23, 37, 6, 24, 34, 21.

- Resolution:
  Items 39, 40, 7, 27, 29, 17, 16, 33, 3, 8, 38, 1, 42.
